# ‘Burden to others’ as a public concern in advanced cancer: a comparative survey in seven European countries

**DOI:** 10.1186/1471-2407-13-105

**Published:** 2013-03-08

**Authors:** Claudia Bausewein, Natalia Calanzani, Barbara A Daveson, Steffen T Simon, Pedro L Ferreira, Irene J Higginson, Dorothee Bechinger-English, Luc Deliens, Marjolein Gysels, Franco Toscani, Lucas Ceulemans, Richard Harding, Barbara Gomes

**Affiliations:** 1 Department of Palliative Care, Policy & Rehabilitation, Cicely Saunders Institute, King’s College London, London, UK; 2 Interdisciplinary Centre for Palliative Medicine, University Hospital Munich, Munich, Germany; 3 Deutsche Gesellschaft für Palliativmedizin, Berlin, Germany; 4 Institute of Palliative Care (IPAC), Oldenburg, Germany; 5 Department of Palliative Medicine, University Hospital Cologne, Cologne, BMBF 01KN1106, Germany; 6 Centre for Health Studies and Research, University of Coimbra (CEISUC), Coimbra, Portugal; 7 End-of-Life Care Research Group, Ghent University & Vrije Universiteit Brussel, Brussels, Belgium; 8 Barcelona Centre for International Health Research (CRESIB – Hospital Clínic), Universitat de Barcelona, Barcelona, Spain; 9 Istituto di Ricerca in Medicina Palliativa, Fondazione Lino Maestroni -ONLUS, Cremona, Italy; 10 University Antwerp, Antwerp, Belgium; 11 Department of Public and Occupational Health, EMGO Institute for Health and Care Research, Palliative Care Center of Expertise, VU University Medical Center, Amsterdam, the Netherlands

## Abstract

**Background:**

Europe faces an enormous public health challenge with aging populations and rising cancer incidence. Little is known about what concerns the public across European countries regarding cancer care towards the end of life. We aimed to compare the level of public concern with different symptoms and problems in advanced cancer across Europe and examine factors influencing this.

**Methods:**

Telephone survey with 9,344 individuals aged ≥16 in England, Flanders, Germany, Italy, Netherlands, Portugal and Spain. Participants were asked about nine symptoms and problems, imagining a situation of advanced cancer with less than one year to live. These were ranked and the three top concerns examined in detail. As ‘burden to others’ showed most variation within and between countries, we determined the relative influence of factors on this concern using GEE and logistic regression.

**Results:**

Overall response rate was 21%. Pain was the top concern in all countries, from 34% participants (Italy) to 49% (Flanders). Burden was second in England, Germany, Italy, Portugal, and Spain. Breathlessness was second in Flanders and the Netherlands. Concern with burden was independently associated with age (70+ years, OR 1.50; 95%CI 1.24-1.82), living alone (OR 0.82, 95%CI 0.73-0.93) and preferring quality rather than quantity of life (OR 1.43, 95%CI 1.14-1.80).

**Conclusions:**

When imagining a last year of life with cancer, the public is not only concerned about medical problems but also about being a burden. Public education about palliative care and symptom control is needed. Cancer care should include a routine assessment and management of social concerns, particularly for older patients with poor prognosis.

## Background

Europe is facing an enormous public health and clinical challenge with aging populations [1] and rising cancer incidence [2]. Cancer treatment has advanced over the last decades, which means that patients live longer experiencing more co-morbidities [3]. Epidemiological changes and technological advances increasingly influence how the public perceives advanced cancer, death, and dying. For example, news reports about cancer frequently discuss aggressive treatment and survival but rarely treatment failure, adverse events, end-of-life care, or death [4].

Public views are reflected in each person diagnosed with cancer. Although clinicians responsible for breaking bad news to patients and families are aware of risks, symptoms and problems associated with various cancers and their treatments, there is little evidence to guide them on what the level of public understanding is [5]. This is important to ensure appropriate communication from the point of diagnosis. In about 50% of cases, the person will not survive from cancer [6]. Communication is then even more difficult. A well informed clinician will know that symptom burden in advanced cancer is high, with pain, breathlessness, and fatigue occurring in over 50% of patients [7]. They will also know that impeccable assessment and advanced symptom management grounded on palliative care will control most symptom discomfort [8]. However, there is a dearth of research to show clinicians the main concerns of people thinking of a situation of advanced cancer. The few existing studies suggest that the public perceived cancer as an extremely painful disease [9], that pain and symptom control comprise main needs in terminal illness [10], and that saying goodbye to loved ones and dying with dignity are essential for a good death [11]. No study has compared public opinion between countries to understand cultural differences. Cross-national research into this topic is important to inform European end of life care policy, education, and research [12].

This study aimed to compare the level of public concern with different symptoms and problems in advanced cancer across varied European countries, and examine factors influencing this.

## Methods

### Design

Population-based telephone survey in seven European countries. Details are described elsewhere [13].

### Participants and settings

The survey was conducted in Flanders (Dutch-speaking part of Belgium), England, Germany, Italy, the Netherlands, Portugal and Spain. The countries were chosen as they participated in a European collaborative (PRISMA) with the aim to promote best practice in the measurement of end-of-life care, setting an agenda and guidance that reflects European cultural diversity, and is informed by both public and clinical priorities [14].

Residents ≥16 years were invited to take part in a computer-assisted telephone interview (CATI). Private households were selected using random digit dialling (RDD), a method to generate a random sample of telephone numbers. The sampling frame was obtained via well-established sampling organisations with a proven record of successfully supplying random samples of telephone numbers to the research industry for over 15 years. The organisations were selected via a strict tendering process and followed a technical specification of work in order to adhere to exacting all methodological, quality and ethical aspects specified by the research team. No quotas (geographic or socio-demographic) were used for sample generation.

Exclusion criteria were incapacity to understand the information and provide informed consent (assessed by interviewers), and inadequate language skills of the country’s dominant language.

### Questionnaire

The questionnaire was developed using a multi-method approach to enhance validity and comparability. This included a review of studies on preferences and priorities for care in advanced cancer, a review of cross-national surveys, and three consultation rounds with 27 palliative care experts. The questionnaire contained 28 questions on preferences and personal values related to care in a scenario imagining *‘a situation of serious illness, for example cancer, with less than one year to live’*. Participants were also asked about their experience with illness, death and dying, their general health and socio-demographics.

One survey component assessed participants’ level of concern regarding nine symptoms and problems which have been chosen based on the above mentioned multi-method approach (see Table 1).

**Table 1:** Survey question about most concerning symptoms and problems

Which of the following nine symptoms or problems do you think would concern you the most?		
A. So which of the following nine symptoms or problems do you think would concern you most?		
B. And in second place?		
**List of problems**	1^st^ place	2^nd^ place
	(1^st^ most concerning)*	(2^nd^ most concerning)*
1. Having no energy		
2. Being in pain		
3. Changes in the way you look		
4. Having no appetite at all		
5. Being a burden to others		
6. Being unable to get your breath		
7. Being alone		
8. Feeling as if you want to be sick		
9. Being worried and distressed		

* scoring: 2 = first most concerning; 1 = second most concerning; 0 = if not selected as first or second most concerning.

### Translation and testing

A formal linguistic process included translation in a systematic and culturally sensitive way into the countries’ dominant language. Following the EORTC translation procedure, forward and backward translations were prepared by two independent native-speakers knowing about end-of-life care and a professional translator [15]. All language versions were harmonized through discussion of country representatives and the final questionnaire was tested and piloted in England and Germany with 30 volunteers using cognitive interviewing [12].

### Procedures

The interviews were conducted by 149 trained interviewers experienced in telephone surveys on social and health issues from May to December 2010. As part of the questionnaire, participants were asked which of nine named symptoms or problems would concern them the most and which would concern them in second place (first/second concern) (Table 1). Interviewers ensured that at least four call-backs were made at different times of the day (with at least one after 6 p.m.) to attempt to reach all potential participants. 10% of interviews were checked by in-situ supervisors for accuracy and interviewer performance, and the research team randomly audited the interviews in real-time.

### Statistical analyses

Sample characteristics were described using crude percentages. First, we derived individual concern scores for each of the nine symptoms and problems and ranked them within and between countries. We then described the three greatest concerns across all countries in more detail.

Second, we determined which of the top three concerns showed most variation to identify influencing factors. Although pain was of most concern, burden showed more variation within and between countries and less consistency than pain. Thus, we then carried out a more detailed analysis of factors associated with choosing burden as a top concern. We compared crude percentages of participants for whom burden was a top concern (first/second concern) with those who ranked it “not most concerning” (i.e. neither first nor second most concerning) and tested for differences in bivariate analyses using *t*-test for age and *χ*^2^-tests and Mann–Whitney U tests for other variables.

To examine factors associated with choosing burden as a top concern across countries we used generalised estimating equations (GEE). This modelling technique takes into account clustering effect within countries, assuming that participants from one country are more likely to have similar views compared to participants from other countries [16]. We entered significant variables from bivariate analysis associated with the concern about being a burden (first/second most concerning versus neither first nor second most concerning, p ≤ 0.05) in the GEE model, using data from all countries where the direction of effect was consistent across countries and also significant data from individual countries. We estimated the odds ratio (OR) associated with different levels of each independent variable retained in the final model (ORs are presented with 95% CIs).

Finally, we conducted logistic regressions within each country, entering factors from the cross-national GEE model (to confirm applicability to individual countries) and other country-specific factors associated with this concern in the bivariate analysis (p ≤ 0.05).

We undertook all analyses using SPSS for Windows (version 19.0.0, 2010; SPSS, Inc, an IBM Company, Chicago, IL). Tests were two-tailed and p ≤ 0.01 was deemed significant in the final models to allow for multiple testing.

### Ethics approval

The study was approved by the research ethics committee of King’s College London, the lead academic centre (BDM/08/09-48). Local research ethics approvals and/or notifications to relevant national data protection agencies were obtained in all countries.

## Results

9,344 people (21%) agreed to participate from 45,242 randomly selected households. The response rate varied across countries, being highest in Germany (29%) and Portugal (28%), followed by Spain, Italy and England (21% each), with Flanders and the Netherlands (each 16%) lowest. Interview completion time averaged 15.4 min (range 3 to 91 min). Main specified reasons for refusal were lack of interest (59%), lack of time (17%) and refusal to take part in telephone surveys (3%). A detailed description for reasons to refuse to take part is available elsewhere [13].

Mean age was 50.7 years, 66% were female and 17% were living alone varying from 24% in England to 11% in Italy and Portugal. 64% described themselves as being religious or belonging to a denomination, ranging from 46% in the Netherlands to 82% in Italy. Ten percent of the participants had been seriously ill in the past five years, and 53% had cared for a close relative or friend in their last months of life (Table 2).

**Table 2:** Participant characteristics by country

	**England**	**Flanders**	**Germany**	**Italy**	**Netherlands**	**Portugal**	**Spain**	**All countries**
	**N = 1351**	**N = 1269**	**N = 1363**	**N = 1352**	**N = 1356**	**N = 1286**	**N = 1367**	**N = 9344**
**Age**								
Mean in years (SD)	54.18 (16.27)	52.18 (14.27)	47.06 (15.71)	48.67 (15.92)	54.53 (14.62)	50.10 (16.85)	48.08 (16.45)	50·68 (16·00)
16-29	8.0%	7.5%	15.8%	15.0%	4.7%	13.8%	15.4%	11.5%
30-39	11.3%	10.2%	14.6%	14.1%	9.7%	14.4%	16.1%	12.9%
40-49	19.1%	22.3%	26.8%	20.4%	22.3%	18.9%	21.0%	21.6%
50-59	19.4%	27.0%	20.3%	23.1%	24.2%	20.1%	22.2%	22.2%
60-69	23.8%	21.9%	13.7%	17.7%	23.6%	18.5%	14.9%	19.1%
70+	18.3%	11.0%	8.8%	9.7%	15.4%	14.3%	10.5%	12.6%
**Gender**								
Female	63.9%	65.6%	58.0%	72.0%	65.8%	69.4%	68.4%	66.1%
**Living arrangements**								
Living alone	24.2%	15.6%	20.8%	10.5%	21.8%	10.6%	11.5%	16.5%
**Urbanisation level**								
Big city or suburbs/outskirts	37.1%	22.8%	40.9%	19.9%	26.8%	50.0%	23.7%	31.5%
Town or small city	36.7%	17.7%	30.8%	39.0%	23.0%	28.6%	43.1%	31.4%
Country village	21.3%	46.6%	22.1%	38.6%	42.7%	17.2%	29.4%	31.1%
Farm or home in countryside	4.9%	13.0%	6.2%	2.5%	7.6%	4.2%	3.8%	6.0%
**Marital status**								
Married or with a partner	61.3%	75.7%	58.1%	63.8%	69.2%	63.6%	62.2%	64.8%
Divorced or separated	13.1%	8.0%	11.3%	6.4%	8.2%	7.1%	7.3%	8.8%
Widowed	9.8%	7.6%	6.2%	6.8%	10.5%	8.5%	8.3%	8.3%
Single	15.8%	8.8%	24.5%	23.0%	12.0%	20.7%	22.1%	18.2%
**Religion**								
With a religion or denomination	57.9%	52.9%	57.0%	81.6%	45.6%	79.6%	71.0%	63.6%
**Health**								
Very good	42.0%	38.6%	22.9%	22.6%	22.3%	13.3%	21.5%	26.1%
Good	39.8%	46.0%	51.5%	47.6%	55.3%	38.1%	41.1%	45.7%
Fair	14.2%	13.9%	21.3%	28.0%	18.8%	43.6%	32.0%	24.5%
Bad	3.5%	1.3%	3.9%	1.6%	3.2%	3.6%	4.9%	3.1%
Very bad	0.5%	0.2%	0.4%	0.2%	0.4%	1.5%	0.5%	0.5%
**Experience of illness, death and dying**								
Diagnosed with seriously illness in last 5 years	12.8%	15.2%	8.0%	8.4%	10.1%	7.8%	8.8%	10.1%
Close relative/friend seriously ill in last 5 years	63.1%	60.6%	64.1%	67.4%	71.8%	57.5%	68.2%	64.8%
Death of close relative/friend in last 5 years	70.6%	69.9%	69.4%	69.3%	76.7%	60.9%	74.4%	70.3%
Cared for close relative/friend in last months of life	50.6%	49.9%	48.0%	60.8%	52.0%	53.2%	57.0%	53.1%

Legend: Sums may not always amount to the total sample number because of missing values on variables. Percentages may not always add up to 100 because of rounding. SD = standard deviation.

### Top concerning symptoms

**Figure 1:**
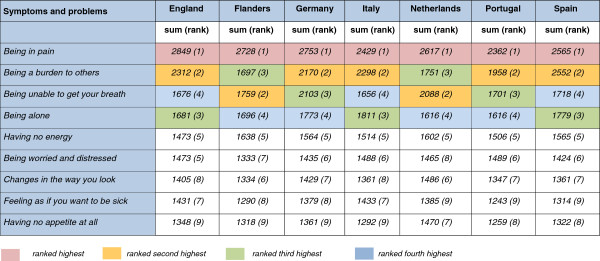
**First and second most important concerns of European citizens by country.** Legend: Sum score: sum of all first most concerning (score = 2) and second most concerning problems (score = 1).

**Figure 2:**
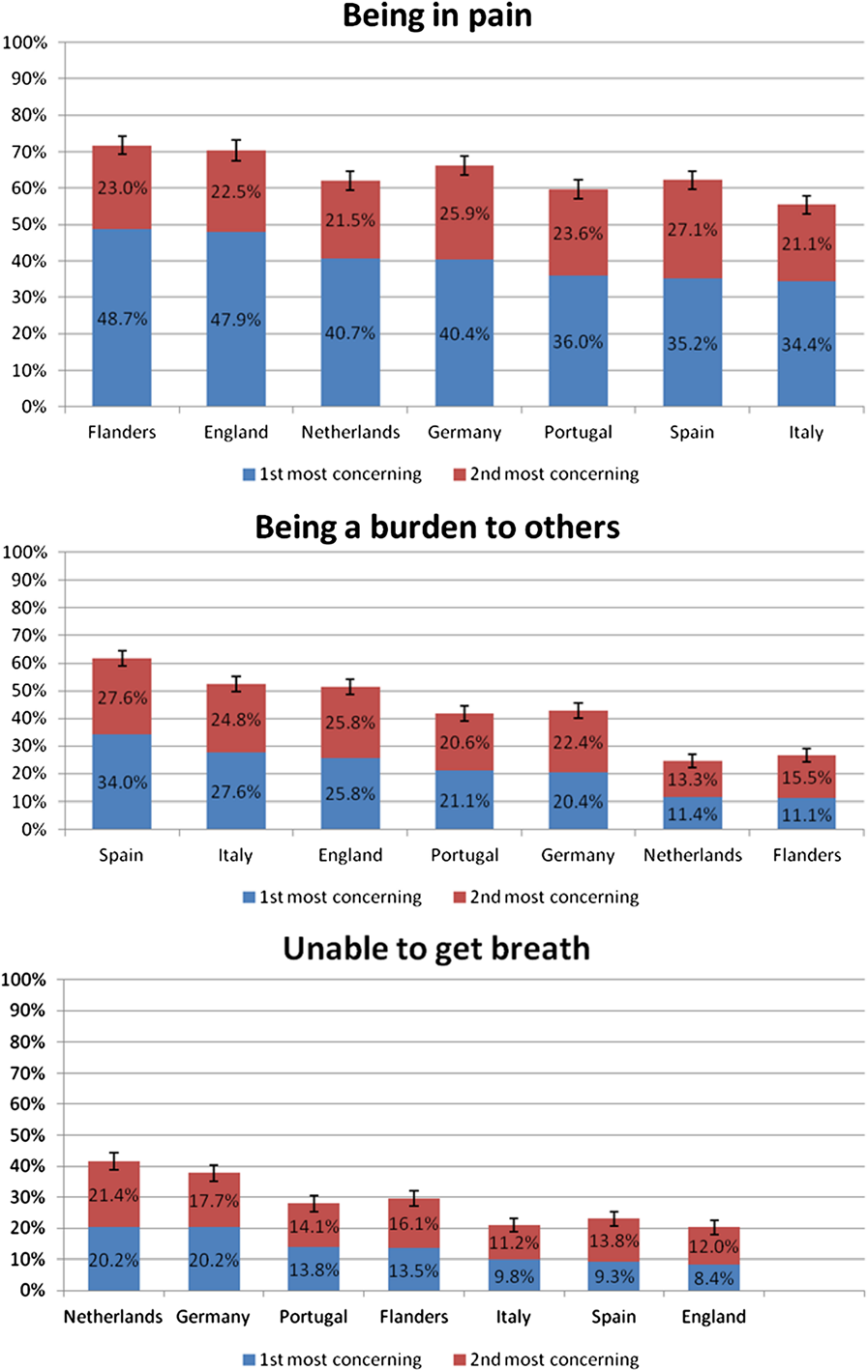
Level of concern for pain, being a burden, and breathlessness (including 95% CI (black line) for combined first and second most concerning) across seven European countries (ranked by first most concerning).

In all seven countries, pain was the top concern for 34% of participants in Italy to 49% in Flanders (Figures 1 and 2). ‘Being a burden’ was the second concern in Spain (34%), Italy (28%), England (26%), Germany, and Portugal, but not in the Netherlands and Flanders where breathlessness was ranked second. In the other five countries, breathlessness and ‘being alone’ ranked third or fourth place.

### Factors influencing concern with ‘being a burden’

Detailed bivariate analyses on concern with ‘being a burden’ are presented in Table 3. Across all countries, concern with burden showed a u-shape relationship with age; it was more frequent among younger (16–29 years; 43.9%) and older people (70+ years; 48.1%) with the lowest point among 40–49 year olds (40.2%; z = 2.347, p = 0.019). In the whole sample, those living alone were more often concerned with burden than those living with others (43.9% vs. 39.7% respectively, *χ*^2^ = 8.43, p = 0.004) but at a country level this effect was significant only in England (53.4% vs.45.6%; *χ*^2^ = 5.75, p = 0.016); in Portugal it was observed the opposite (31.9% vs. 42.9%; *χ*^2^ = 5.20, p = 0.023). Perceptions of one’s own health and personal experiences of illness, death and dying did not affect concern with burden. In the whole sample, burden concern was highest amongst participants who preferred care on improving the quality rather than the quantity of life across all countries (*χ*^2^ = 18.80, p = <0.0001); this also reached significance in Germany (*χ*^2^ = 7.1, p = 0.03) and Italy (*χ*^2^ = 10.1, p = 0.006) but not in other countries.

**Table 3:** **Proportion of respondents rating “being a burden to others” as 1**^**st **^**or 2**^**nd **^**most concerning problem (crude percentages by variables of interest)**

	**England**	**Flanders**	**Germany**	**Italy**	**Netherlands**	**Portugal**	**Spain**	**All countries**
	**n = 1351**	**n = 1269**	**n = 1363**	**n = 1352**	**n = 1356**	**n = 1286**	**n = 1367**	**n = 9344**
**Age**		**	*		**		**	*
Mean age of people who are concerned of being a burden (vs. others)	54.1 vs. 53.5	54.0 vs 51.3	48.1 vs 46.1	48.7 vs 47.3	56.4 vs 53.3	49.3 vs 48.7	48.7 vs 46.2	50.7 vs. 49.8
Age bands		**	*		**		*	*
16–29	52.8%	20.7%	42.7%	47.2%	23.0%	39.6%	57.7%	43.9%
30–39	52.7%	16.2%	39.1%	56.3%	19.7%	42.4%	58.0%	42.8%
40–49	51.4%	22.1%	38.2%	54.5%	18.7%	37.6%	62.4%	40.2%
50–59	47.3%	28.6%	45.5%	50.2%	24.8%	42.9%	63.0%	42.6%
60–69	50.2%	27.8%	48.1%	60.6%	26.8%	41.4%	64.0%	44.1%
70+	57.5%	31.1%	48.7%	52.5%	33.0%	44.1%	69.4%	48.1%
**Gender**			**					*
Male	53.8%	26.2%	47.7%	53.1%	24.0%	44.4%	63.6%	44.7%
Female	50.2%	26.8%	39.2%	52.1%	25.0%	40.6%	60.7%	42.5%
**Living arrangements**	*					*		**
Living alone	53.4%	26.1%	43.1%	52.7%	24.7%	31.9%	61.3%	43.9%
Living with others	45.6%	28.2%	41.3%	50.0%	24.9%	42.9%	63.4%	39.7%
**Urbanisation level**								**
Big city or suburbs/outskirts	53.6%	25.8%	41.3%	55.5%	22.9%	43.3%	61.6%	43.6%
Town or small city	51.7%	30.0%	40.9%	50.2%	24.2%	39.8%	61.7%	45.8%
Country village	47.5%	24.9%	46.0%	54.6%	24.8%	41.1%	62.2%	40.9%
Farm or home in countryside	53.1%	29.7%	50.0%	31.3%	31.6%	38.8%	56.5%	39.4%
**Marital status**	*						**	
Married or with partner	54.4%	25.9%	43.6%	54.1%	24.2%	43.2%	64.4%	43.6%
Divorced or separated	47.0%	34.7%	43.4%	52.5%	28.0%	36.8%	59.4%	43.3%
Widowed	52.5%	29.8%	50.0%	55.8%	31.3%	35.6%	66.3%	45.6%
Single	44.0%	22.9%	37.7%	46.8%	20.4%	41.4%	53.1%	40.9%
**Religion**								**
Yes	51.3%	28.2%	42.3%	52.4%	22.9%	41.1%	60.9%	44.5%
No	51.9%	25.2%	43.3%	51.8%	26.1%	44.6%	63.3%	40.9%
**Education**		*						**
Less than primary education	52.8%	16.0%	0%	66.7%	23.8%	36.4%	60.7%	49.8%
Primary to secondary education	53.2%	23.9%	43.6%	53.7%	25.3%	41.0%	64.1%	43.9%
Post secondary to tertiary education	49.0%	29.1%	41.6%	48.8%	23.8%	44.1%	57.9%	41.2%
**Activities in last seven days**								
Paid work					*			**
*Yes*	53.3%	25.8%	42.2%	51.4%	21.9%	42.2%	61.5%	41.8%
*No*	50.1%	27.5%	43.3%	53.1%	28.0%	41.3%	61.7%	44.6%
In education								
*Yes*	50.6%	24.2%	39.8%	46.6%	33.3%	39.8%	56.6%	42.6%
*No*	51.7%	26.8%	43.0%	53.0%	24.2%	41.9%	62.3%	43.3%
Retired								
*Yes*	51.2%	29.5%	46.0%	55.6%	28.5%	40.7%	63.9%	44.5%
*No*	51.8%	25.5%	41.7%	51.3%	23.5%	42.1%	61.1%	42.8%
**Financial hardship**		*						**
Living comfortably on present income	50.8%	29.0%	42.3%	50.4%	26.2%	43.5%	60.1%	40.8%
Coping on present income	53.8%	24.5%	43.1%	55.8%	23.0%	43.3%	64.8%	45.6%
Difficult on present income	46.2%	17.2%	46.4%	48.2%	17.5%	38.4%	57.1%	43.4%
Very difficult on present income	54.3%	0%	37.5%	31.6%	25.0%	38.1%	55.7%	42.6%
**Health**								
Very good	52.2%	28.8%	43.4%	46.1%	25.3%	44.2%	59.6%	42.6%
Good	52.5%	24.9%	41.2%	54.3%	25.0%	41.1%	63.3%	42.4%
Fair	46.2%	25.0%	47.5%	54.3%	25.4%	41.3%	62.0%	45.6%
Bad	54.3%	33.3%	39.2%	50.0%	11.9%	50.0%	58.1%	43.8%
Very bad	28.6%	33.3%	0%	66.7%	25.0%	29.4%	28.6%	28.3%
**Experience of illness, death and dying**								
Diagnosed with seriously illness in last 5 years								
*Yes*	52.1%	23.6%	46.6%	49.5%	27.5%	40.9%	60.5%	41.7%
*No*	51.5%	27.1%	42.3%	52.5%	24.3%	41.9%	61.8%	43.3%
Close relative/friend seriously ill in last 5 years								
*Yes*	52.5%	26.9%	41.6%	52.5%	25.1%	40.9%	60.6%	43.1%
*No*	49.7%	25.9%	44.4%	51.4%	23.5%	43.4%	64.2%	43.3%
Death of close relative/friend in last 5 years								
*Yes*	50.7%	27.1%	43.9%	51.0%	25.5%	43.8%	61.8%	43.4%
*No*	53.1%	25.2%	40.1%	55.1%	21.9%	38.6%	61.8%	42.6%
Cared for relative/friend in last months of life		*						
*Yes*	50.7%	29.4%	43.6%	52.0%	26.2%	39.8%	62.6%	44.1%
*No*	52.3%	24.0%	42.1%	52.9%	23.0%	44.2%	60.7%	42.3%
**Quality or quantity of life**			*	**				**
To extend life	37.0%	23.8%	30.0%	51.4%	22.9%	31.4%	50.0%	34.1%
Both equally important	49.6%	22.5%	38.4%	46.7%	21.3%	41.0%	60.2%	41.1%
To improve quality of life	52.8%	27.7%	44.9%	55.9%	25.8%	43.1%	62.2%	44.4%

Legend: Significant results from bivariate analysis are indicated by * (p < 0.05) and ** (p < 0.01).

The final cross-national GEE model consisted of three factors (age, living alone and quality/quantity of life) independently associated with burden concern (Table 3). Gender, education, paid work in last seven days, and financial hardship were entered but not retained as they failed to reach significance in the presence of other factors. Urbanization level and religion were not included as there were no significant associations on a country level. Of the six variables, only age, living alone and emphasis on quality of life remained significant (Table 4).

**Table 4:** Factors associated with choosing being a burden to others as top concern in cross-national and national models (GEE and logistic regression)

	**All countries (GEE)**	**Germany**^a^	**Portugal**^a^	**Spain**^a^
	**n = 9344**	**n = 1208**	**n = 1101**	**n = 1226**
**Cross-National Variables**	OR	OR	OR	OR
	(95% CIs)	(95% CIs)	(95% CIs)	(95% CIs)
**Age (ref 16–29)**				
Age bands				
30–39	1.02 (0.86–1.22)	n.s.	n.s.	n.s.
40–49	1.01 (0.86–1.18)	n.s.	n.s.	n.s.
50–59	1.13 (0.97–1.33)	n.s.	n.s.	n.s.
60–69	1.26 (1.07–1.49)	n.s.	n.s.	n.s.
70+	1.50 (1.24–1.82)	n.s.	n.s.	n.s.
**Gender (ref male)**				
Female	n.s.	0.69 (0.55–0.88)	n.s.	n.s.
**Living arrangements (ref living with others)**				
Living alone	0.82 (0.73–0.93)	n.s.	0.60 (0.39–0.93)	n.s.
**Quality or quantity of life (ref to extend life)**				
Both equally important	1.17 (0.92–1.50)	1.52 (0.79–2.93)	n.s.	n.s.
To improve quality of life	1.43 (1.14–1.80)	1.96 (1.05–3.66)	n.s.	n.s.
**Country Specific Variable**				
**Marital status (ref being married)**				
Divorced/separated	n.s.	n.s.	n.s.	0.75 (0.47–1.19)
Widowed	n.s.	n.s.	n.s.	0.91 (0.53–1.59)
Single	n.s.	n.s.	n.s.	0.55 (0.38–0.80)

Legend: Only countries with significant variables are presented (p ≤ 0.01); ^a^ logistic regression.

The ORs for being concerned with burdening others increased with age and were highest in the 60–69 and 70+ groups. Once age was taken into account, people living alone were less likely to be concerned with burdening others. Also, ORs were higher for people who preferred care to focus on quality rather than quantity of life.

Distinctions between countries revealed that in Germany, women were less likely to be concerned with burden, and wishing quality rather than quantity of life had a stronger independent influence. In Portugal, living alone had relatively less influence on concern with burdening others.

## Discussion

This is the first cross-national survey assessing concerns of the public when considering advanced cancer in the last year of life. In all seven European countries examined, being in pain, a burden to others or being breathless ranked highest. The concern with burden showed most variation within and between countries. Older age, living alone, and a preference to improve quality rather than quantity of life accounted for some of this variation. Older age and a wish for quality increased the concern with burden, whereas living alone decreased it.

These results are based on a sound cross-national comparison using standard methodologies and asking identical questions across countries. Thus, the findings provide invaluable and rare information for national and international practice and policy indicating that more education and research should focus on the top concerns being a burden and being in pain.

There are also some limitations; the response rate although low is similar to the declining rates of RDD surveys [17]. Furthermore, there are selection biases; those without access to a fixed telephone (29% of households in the EU-27) [18] were excluded, and women and older people are over-represented, due to selective non-response. We were not able to obtain more information from non-respondents due to the nature of the survey (random selection of telephone numbers), although we know the main reasons why people did not take part in the study (majority due to lack of interest and lack of time). The bias towards older people might have an impact on our findings as older age was an independent factor predicting being concerned with burden in all participants (although the influence of age was not confirmed on a national level). Therefore, considering all countries together it is possible that choosing a burden as a top concern was overestimated in our sample. The bias toward women might have had an impact in Germany, as in this country women were found to be less likely to choose burden as a top concern. In this case, the concern about being a burden might be higher for the German population than it was in our sample.

### Pain

Despite advances in pain management over the last decades, nearly one in two cancer patients suffer from unrelieved pain and the prevalence is higher in advanced stages [19-21]. Thus, it is not surprising that the public is most concerned about pain when imagining advanced cancer. Varied perceptions of pain might have influenced the answers. Half of the participants had previous experience of caring for a close relative/friend and might have memories related to pain. Although often understood as a primarily physical sensation, pain could be a substitute for suffering and distress especially as a cancer diagnosis evokes images of pain, suffering, and death [22]. Public perception seems unchanged over the last 25 years, when cancer was considered to be an extremely painful disease relative to other medical conditions [9].

Concerns about pain showed a clear north–south gradient with respondents from Southern Europe being less concerned than their northern counterparts. Differences may exist between more secular Western European societies and more religious societies as in Southern Europe with a predominantly Roman Catholic tradition where acceptance of suffering, with physical pain may be perceived as a prototype, is thought to foster spiritual growth [23-25]. A lower opioid consumption in Southern European countries compared to Western Europe also reflects this [26] as well as fear that opioids may impair cognitive function and hasten death [23].

### Being a burden

Self-perceived burden is thought to be a universal concern across countries, important for achieving a good death [5,10]. However, our survey showed variation with more than half of the respondents expressing this concern in Spain, Italy, and England in contrast to 25% from the Netherlands and Flanders. Previous research shows that self-perceived burden affects patients’ well-being; for example, it is associated with hopelessness, quality of life, and depression [27]. In end-of-life care situations, self-perceived burden has been found to underlie the choice for institutionalization [28] and request for euthanasia [29,30].

Older age was a predictor for concerns with burden. This concurs with other results but on a cross-national level [11,31]. The implications are important in the context of ageing populations and as the cancer trajectory increases in length, with more potential to ‘burdening others’. The consequences are varied. For example, fear of being a burden has been found to lead older people to prepare for death (e.g. making a will or funeral arrangements) [32]. However, it is also a key factor of the social relationship dynamics which can erode the sense of dignity of nursing home residents [31].

Interestingly, once the effect of age was taken into account those living alone were less concerned with being a burden. People living alone might not have family and others to worry about, they might be more independent and have learnt to live by themselves and sort their problems. Although most people wish to die at home [33], living alone has been one of the factors identified to preclude home death [34]. People living alone might be aware of the higher chance to die in an institution and thus are less worried about being a burden to their significant others.

A considerable proportion of respondents had previous experience with serious illness such as cancer, death and dying giving them a “double” status of being a member of the public and affected either personally or as a career. However, this did not influence the perception of being a burden. Similarly, it did not influence a preference for home death (data published elsewhere) [35].

### Implications for education and clinical practice

Although palliative care has been established across Europe and is now compulsory in many medical schools, education about palliative care and symptom control options does not seem to have reached the general public sufficiently. This has already been postulated 10 years ago [10] but still seems to be topical.

A variety of factors leads to undertreatment of cancer pain with fear of patients (e.g. to become addicted) to utilize opioids being one of them [36]. It is therefore important for clinicians to know the expectations and concerns of patients and family carers and to provide sufficient information about pain management and opioids.

The concern of self-perceived burden has important implications for the provision of cancer care towards the end of life. First, it highlights the need for a holistic approach rather than a medicalization of care. Cancer care should include a routine assessment and management of social concerns, particularly for older patients with poor prognosis. Second, it raises questions regarding policy making. In many European countries, there is a trend towards end-of-life care at home and in the community. This will result in a heavier share of care on family carers while their availability is diminishing due to changing populations, smaller families and the increasing number of women choosing employment over caring tasks. Therefore, self-perceived burden by patients and its detrimental consequences will need to be addressed by better support for family carers and better home care.

## Conclusions

Main public concerns for the last year of life are pain, being a burden, and breathlessness. More public education is needed to inform people about the potential of palliative care but also about the non-medical aspects of end-of-life care. Clinicians should always explore concerns of patients and relatives to better understand their perceptions and fears.

## Competing interests

The authors declare that they have no competing interests.

## Authors’ contributions

All authors contributed to study design, survey development, data analysis, and took part in the interpretation of findings and drafting of the manuscript. BG and NC coordinated the development and implementation of the computer-assisted telephone interview by BMG Research and ZEM University of Bonn. BAD aided this process and the commissioning of the study. BAD, STS, CB, BG, RH and DBE conducted the survey pilot. CB conducted the analysis of the symptoms data supervised by BG. NC aided data management throughout the period of data analysis, and prior to this. IJH and RH helped to conceive the idea for the study, collaborated in its design and interpretation. CB took the main responsibility for writing the manuscript and the concept of this paper. STS and PLF aided the initial development of the idea behind this paper. All authors helped notably with survey construction, and cultural adaptation of the survey and the interpretation of its findings. All authors read and approved the final manuscript.

## Authors’ information

PRISMA Members: Gwenda Albers, Barbara Antunes, Emma Bennett, Ana Barros Pinto, Claudia Bausewein, Dorothee Bechinger-English, Hamid Benalia, Lucy Bradley, Lucas Ceulemans, Barbara A Daveson, Luc Deliens, Noël Derycke, Martine de Vlieger, Let Dillen, Julia Downing, Michael Echteld, Natalie Evans, Dagny Faksvåg Haugen, Nancy Gikaara, Barbara Gomes, Marjolein Gysels, Sue Hall, Richard Harding, Irene J Higginson, Stein Kaasa, Jonathan Koffman, Pedro Lopes Ferreira, Johan Menten, Natalia Monteiro Calanzani, Fliss Murtagh, Bregje Onwuteaka-Philipsen, Roeline Pasman, Francesca Pettenati, Robert Pool, Tony Powell, Miel Ribbe, Katrin Sigurdardottir, Steffen Simon, Franco Toscani, Bart van den Eynden, Jenny van der Steen, Paul Vanden Berghe, Trudie van Iersel.

## Pre-publication history

The pre-publication history for this paper can be accessed here:

http://www.biomedcentral.com/1471-2407/13/105/prepub
